# Adeno-Associated Viral Vectors in the Treatment of Epilepsy

**DOI:** 10.3390/ijms252212081

**Published:** 2024-11-11

**Authors:** Aysilu I. Mullagulova, Elena E. Timechko, Valeriya V. Solovyeva, Alexey M. Yakimov, Ahmad Ibrahim, Diana D. Dmitrenko, Albert A. Sufianov, Galina Z. Sufianova, Albert A. Rizvanov

**Affiliations:** 1 Institute for Fundamental Medicine and Biology, Kazan Federal University, Kazan 420008, Russia; ayimullagulova@kpfu.ru (A.I.M.); vavsoloveva@kpfu.ru (V.V.S.); akhmibrakhim@kpfu.ru (A.I.); 2 Department of Medical Genetics and Clinical Neurophysiology, Krasnoyarsk State Medical University, Partizana Zheleznyaka 1, Krasnoyarsk 660022, Russia; e.e.timechko@yandex.ru (E.E.T.); cilvana20010z@gmail.com (A.M.Y.); mart2802@yandex.ru (D.D.D.); 3 Department of Neurosurgery, Sechenov First Moscow State Medical University of the Ministry of Health of the Russian Federation (Sechenov University), Moscow 119991, Russia; sufianov_a_a@staff.sechenov.ru; 4 The Research and Educational Institute of Neurosurgery, Peoples’ Friendship University of Russia, Moscow 117198, Russia; 5 Department of Pharmacology, Tyumen State Medical University, Tyumen 625023, Russia; sufarm@mail.ru; 6 Division of Medical and Biological Sciences, Academy of Sciences of the Republic of Tatarstan, Kazan 420111, Russia

**Keywords:** adeno-associated virus, epilepsy, gene therapy, neuropeptides, ion channels, receptors, transcription factors, neurotrophic factors, antisense oligonucleotides

## Abstract

Epilepsy is a brain disorder characterized by a persistent predisposition to epileptic seizures. With various etiologies of epilepsy, a significant proportion of patients develop pharmacoresistance to antiepileptic drugs, which necessitates the search for new therapeutic methods, in particular, using gene therapy. This review discusses the use of adeno-associated viral (AAV) vectors in gene therapy for epilepsy, emphasizing their advantages, such as high efficiency of neuronal tissue transduction and low immunogenicity/cytotoxicity. AAV vectors provide the possibility of personalized therapy due to the diversity of serotypes and genomic constructs, which allows for increasing the specificity and effectiveness of treatment. Promising orientations include the modulation of the expression of neuropeptides, ion channels, transcription, and neurotrophic factors, as well as the use of antisense oligonucleotides to regulate seizure activity, which can reduce the severity of epileptic disorders. This review summarizes the current advances in the use of AAV vectors for the treatment of epilepsy of various etiologies, demonstrating the significant potential of AAV vectors for the development of personalized and more effective approaches to reducing seizure activity and improving patient prognosis.

## 1. Introduction

Epilepsy is a brain disease characterized by a persistent predisposition to the occurrence of seizures. It is diagnosed by at least two unprovoked seizures with an interval of >24 h [1]. Epilepsy syndrome is defined as a characteristic set of clinical and electroencephalographic signs, often supported by specific etiological data (structural, genetic, metabolic, immune and infectious) [2]. According to the International League against Epilepsy (ILAE), epilepsy can be classified into the following types: structural, genetic, infectious, metabolic and immune [3]. However, epilepsy often falls into several categories. The area of origin of the attack in the brain is the basis for classifying epilepsy into focal, generalized and epilepsy with unspecified onset [4].

Regardless of the etiology and type of seizures, epilepsy is characterized by a constant predisposition to unprovoked seizures caused by an imbalance between mediators of excitation and inhibition of neuronal activity [5]. The incidence of epilepsy also depends on age, with the highest rates observed in children under 5 years of age and people over 65 years of age. Diagnosis of epilepsy in infants can be difficult due to the variety of symptoms and their overlap with normal development. Children who have the disease onset at an early age often experience cognitive and behavioral disorders [6]. In adolescence, epilepsy is a significant neurological problem, the prevalence of which ranges from 1.5% to 2%. Puberty is not believed to affect seizure frequency. However, there are studies showing that estrogen can activate epileptiform activity, testosterone can reduce seizure activity, and progesterone can reduce epileptiform discharges [7]. These effects are due to the influence on the transmission of γ-aminobutyric acid (GABA) [8]. Studies of the genetic forms of epilepsy have revealed several pathogenic variants in various genes [9,10,11], most of which encode ion channel subunits, which helped to better study the pathogenesis of the disease and to identify potential targets for gene therapy. However, in some cases, especially for focal epilepsy, the presence of pathogenic variants is uncharacteristic [12,13]. Nevertheless, proteomic and/or transcriptomic profiling demonstrates the aberrant expression of a number of gene transcripts and their products involved in the regulation of excitatory and inhibitory activity [14,15,16].

A characteristic feature, especially for structural forms of epilepsy, is that epileptic seizures are insufficiently stopped or not stopped at all with the help of antiepileptic drugs (AEDs). The development of pharmacoresistance to AEDs is observed in a significant proportion of patients with epilepsy, estimated to be up to one-third of all patients with this disease [17,18]. These facts represent a serious problem in neurology since traditional treatment methods lose their effectiveness, which leads to frequent, difficult-to-control seizures, deterioration in the quality of the patient’s life and an increased risk of complications. It also emphasizes the importance of developing and implementing new therapeutic approaches aimed at overcoming resistance and increasing the effectiveness of epilepsy treatment, in particular, advanced therapy medicinal products (ATMPs). ATMP drugs are innovative, complex therapeutic agents that can potentially be used to treat various diseases, including cancer, neurodegenerative diseases (such as Huntington’s and Parkinson’s diseases), hereditary diseases and autoimmune diseases [19]. ATMPs are usually divided into three categories: (1) gene therapy (drugs for gene therapy); (2) somatic cell therapy [20]; and (3) tissue engineering products, such as vesicles [21,22], as well as any combination of the above. A detailed review of the methods of advanced therapy in the treatment of epilepsy is described in the work of Shaimardanova et al. [23,24].

To date, there are many studies devoted to the development of vector viral systems potentially used in the treatment of diseases of the central nervous system (CNS) [25]. Most of the efforts have been aimed at developing vectors based on the following viruses: adenovirus, lentivirus, adeno-associated virus and herpes simplex virus [26]. Adenoviral vectors, despite their ability to transduce neuronal and glial cells, which are the main targets in the treatment of CNS diseases [27], as well as the ability to carry a large transgenic insert [28], are less suitable for clinical use due to high levels of cellular toxicity. Cells transduced by adenoviruses can be destroyed by cellular immune mechanisms, which, in particular, involve natural killers and cytotoxic T lymphocytes. In addition, adenoviruses are capable of damaging the cell membrane, disrupting normal cellular functions and leading to apoptosis of infected cells [29,30,31]. According to research, the herpes simplex virus is also potentially toxic to cells [32]; in addition, the production of viral particles based on it is a rather laborious process [33]. Lentivirus is a representative of the retrovirus class [34] and, therefore, has the ability to integrate into the genome, which can lead to an increase in the mutational load of the genome in cases of inappropriate embedding of genetic material [35,36].

Adeno-associated virus (AAV) is one of the most appealing viruses for developing vector systems; it is most often used in gene therapy for neurological disorders [37] due to the high transduction ability of neuronal tissues for a variety of serotypes and the absence of toxicity to cells [38]. Attempts to use AAV vectors in the treatment of neurological disorders have been made for a relatively long time [39]. In particular, for a number of disorders classified as lysosomal storage diseases [40], clinical trials were conducted using viral vectors [41], such as Canavan disease [42,43]—a pediatric neurodegenerative disease associated with pathogenic variants in the aspartoacylase gene (*ASPA*); late infantile neuronal ceroid lipofuscinosis (*LINCL*) [44]; and a number of other diseases; a detailed analysis of these materials can be found in a number of publications [45,46,47,48]. The introduction of a targeted gene using AAV to significantly reduce the frequency of seizures and relieve symptoms may be an effective strategy for the treatment of epilepsy. In this study, we analyze the existing achievements in the use of AAV vectors in the treatment of epilepsy of various etiologies.

## 2. Tropism and Transduction Capacity of AAV Vectors

The use of AAV-based vectors is an attractive strategy for gene therapy of neurological disorders. To date, twelve human serotypes (from AAV1 to AAV12) and more than a hundred primate serotypes have been identified [49]. AAV is a small, about 25 nm, nonenveloped virus of the genus *Dependovirus* of the family *Parvoviridae* [50], which contains a linear single-stranded DNA genome containing about 4.8 thousand nucleotides [51]; both sense and antisense DNA strands in equal proportions can be packed into the capsid. Both ends of DNA contain inverted terminal repeats (ITRs) with a length of about 145 nucleotides, and two open reading frames (ORFs) have been found in the genome: *Rep* and *Cap* [52]. ITR regions are palindromic sequences necessary for the effective replication of the AAV genome [53]; they form a secondary hairpin structure at the ends of DNA, which leads to “self-priming” during replication and the possibility of second-strand synthesis without the involvement of primases. In addition, ITR is a necessary cis-regulatory element for the replication and encapsulation of a therapeutic or reporter gene when using AAV in gene therapy [51], while *Rep* and *Cap* can be delivered in trans.

*Rep* ORF encodes Rep proteins. There are three viral promoters named after their localization in the viral genome: p5, p19 and p40 [54,55], the first two of which encode Rep proteins. The p5 and p19 promoters regulate the transcription of two overlapping mRNAs of different lengths, each of which contains an intron that can be spliced. Unspliced mRNAs are translated into the Rep78 and Rep52 isoforms, whereas the Rep68 and Rep40 isoforms are translated from spliced mRNAs [56]; the name of the proteins reflects their size in kDa. All Rep isoforms share a common central domain that has ATPase and DNA helicase activity [57]. Rep68 and Rep78 also have a specific domain capable of binding the terminal resolution site (TRS) within the hairpin formed by the ITR present at the N-terminus of the protein, which has site-specific rupture activity [58]. Rep68 and Rep78 are necessary for AAV DNA replication [59]. The small Rep proteins, Rep40 and Rep52, are necessary for the efficient packaging of the AAV genome into AAV capsids [60].

*Cap* ORF encodes overlapping sequences of three capsid proteins, VP1, VP2 and VP3, and two accessory proteins, membrane-associated accessory protein (MAAP) and associated accessory protein (AAP) [56]. These proteins are essential components of the AAV structure and play crucial roles in the virus’s life cycle. The capsid proteins VP1, VP2 and VP3 are critical for viral capsid formation, protecting the viral genome and mediating genetic material delivery into host cells [61]. VP1 and VP2 are often involved in endosomal trafficking and genome release [62]. As for VP3, it is considered the main structure and can form VP3-only capsids [63]. The presence of these overlapping sequences allows for the coordinated expression of these proteins from a single promoter, specifically the p40 promoter, which initiates transcription. In total, the icosahedral capsid includes about 60 monomers of VP proteins (VP1, VP2, VP3) in a ratio of 1:1:10, respectively [64]. Furthermore, the accessory proteins MAAP and AAP are involved in promoting capsid stability and assembly and enhancing viral infectivity [65,66]. MAAP is associated with the viral membrane and plays a role in AAV replication [65], while AAP assists in the assembly and production of viral particles [66]. A study on AAV2 showed that in the absence of AAP2, the viral proteins VP1, VP2 and VP3 remain unstable, emphasizing the important role of AAP in the AAV life cycle [67].

All three VP proteins are translated from a single mRNA, which can be spliced in three ways by excision of a long or short intron. The CAP gene also produces an additional nonstructural protein, AAP, whose precise function in capsid assembly is not yet clear [68]. The AAV-based expression vector must be co-transfected with a helper plasmid that mediates the expression of the *Rep* and *Cap* genes. In addition, specific adenovirus genes required for AAV replication must be delivered in trans [69].

### 2.1. Features of Application and Effectiveness of AAV Vectors

A variety of naturally occurring AAV serotypes have been described in the literature [70], which, despite high sequence homology, differ in the surface properties of the capsid [49,71,72]. Combining AAV-based genomic constructs with *Cap* genes derived from different AAV serotypes leads to the possibility of creating so-called pseudotyped recombinant AAV (rAVV). Packaging systems for approximately 10 different serotypes are available for vector construction [73].

Efficient transduction depends on the interaction of capsid proteins with cellular receptors, which ensures the endocytosis, transportation and nuclear internalization of viral particles [74]. Thus, a number of studies have described significant differences in the transduction efficiency of different AAV serotypes for specific tissues and cell types [75,76,77,78]. Therefore, the efficient expression of the target gene in certain cells directly depends on the serotype of the virus used. In addition to tropism due to the AAV serotype, the efficient expression of the target gene using AAV vectors can vary significantly, also depending on the type of expression cassette promoter used [79]. The effect of the promoter on tissue specificity and expression capacity was demonstrated in studies using the myelin basic protein promoter [80] and the glial fibrillary acidic protein (GFAP) promoter [81]. Often, cytomegalovirus (CMV), chicken β-actin (CBA) and cytomegalovirus/chicken beta-actin (CBh) promoters are used in the vector cassette [82]. In particular, AAV9 under the control of CBA demonstrated dominant neuronal transduction, while AAV under the control of a truncated CBA–CBh promoter shifted transduction towards oligodendrocytes [83]. In the work of Jackson et al. [84], a more specific neuronal transduction capacity of an AAV vector using the synapsin promoter was demonstrated and compared to the hybrid CBh promoter, which, despite higher expression, has tissue specificity outside the CNS.

Despite their low immunogenicity profile, AAV vectors can stimulate antiviral immune responses directed against the capsid and/or transgene, especially when delivered systemically or at higher vector doses [85]. Neutralizing antibodies (NAbs) against AAV can be produced following exposure to naturally occurring AAVs. An anti-capsid humoral immune response is triggered in patients receiving gene therapy, which subsequently leads to the neutralization of cross-reactive injected AAV [86,87]. As a result, NAb screening is a prerequisite before inclusion in AAV-based clinical trials, with high pre-existing titers being an exclusion parameter. This criterion immediately limits the widespread use of AAV for the treatment of diseases until further optimization creates the opportunity to evade NAb [88]. Immunomodulatory methods are currently being actively developed to reduce the immunogenicity of the AAV vector and ensure safe and repeatable interventions. Corticosteroids are often used in trials and help modulate immune-mediated toxicity and achieve long-term transgene expression [89].

### 2.2. Application of AAV Vectors for Nervous System Transduction

To date, the most studied AAV serotype is AAV serotype 2 (AAV2). Vectors based on AAV serotype 2 are well suited for transferring target genes to the CNS, which has been demonstrated in the studies of Parkinson’s disease [90] and Alzheimer’s disease [91]. According to studies, AAV2 is capable of transducing postmitotic cells [92]. Also, according to Du et al., neurons are highly susceptible to this serotype [93]. Pathogenicity for humans has not been identified using AAV2; in addition, the virus is unable to replicate without the auxiliary functions provided by another virus, such as adenovirus or herpes simplex virus. rAAV2 vectors are less immunogenic than other viral vectors since they do not express any viral genes and are not able to effectively transduce antigen-presenting cells after vector administration in vivo [94]. In animal models, rAAV2 vectors provide long-term stable expression of encoded transgenes in both the brain and retina [95,96].

Preclinical studies using rAAV2 vectors show that following intraparenchymal injection into the brain of animals, transduction occurs predominantly in neurons, with the transduction of other cell types, such as astrocytes and microglia, being rare [75,81,95,97]. Using larger animals to assess transduction capacity, such as *Felis catus* [98] and primates [99,100], have also demonstrated predominantly neuronal transduction of AAV2. Resected human hippocampal tissue that had undergone surgery for refractory temporal lobe epilepsy also showed a strictly neuronal tropism for this serotype [101]. However, one of the problems with using this serotype is that transgene expression following direct injection of the rAAV2 vector into a specific brain region is usually limited to the region around the injection needle site and, accordingly, the virus has a poor ability to spread to more distal regions [98,102]. The efficiency of transduction and expression capacity can be increased by the additional administration of agents that facilitate the binding of the viral capsid to the cellular receptor. For example, methods have been investigated for the additional administration of heparin, which binds to heparan sulfate proteoglycan (HSPG), thereby increasing the efficiency of viral vector binding to CNS cells and promoting more successful transduction [103,104]; fibroblast growth factor (FGF) has a high affinity for HSPG and can interact with heparin-like glycosaminoglycans (HLGAG) of the extracellular matrix (ECM), affecting the efficiency of viral vector binding and penetration [105]; or mannitol [106,107], an osmotic agent that increases the permeability of the blood–brain barrier (BBB), thereby enhancing the distribution of AAV in the CNS [108,109]. In particular, the delivery of AAV vectors to the brain of mice with the prior administration of mannitol to increase BBB permeability was reported [110], which led to greater accumulation of the transgene. However, the use of additional agents is associated with a number of difficulties; for example, a potential risk of bleeding associated with heparin sulfate injection has been described [102]. Another problem with the use of AAV2 is the fact that 18–70% of the population already have neutralizing antibodies to AAV2 [111,112], which may reduce the efficiency of transduction and expression of this serotype vector rAAV2 in the CNS [113,114].

Other AAV serotypes have also been evaluated for their ability to transfer genes to the CNS. In particular, the work by Davidson et al. reported that rAAV vectors derived from serotypes 4 and 5 exhibited increased gene transfer efficiency and broader distribution of transduced cells throughout the brain following direct injection into the mouse striatum compared to serotype 2. Higher transduction capacity for AAV4 and AAV5 was indicated following intraventricular injection compared to AAV2. Intrastriatal injection showed the highest efficiency for AAV5, and its expression, as well as the AAV4 expression, was stable but lost 15 weeks after administration [115]. These findings were also supported by another study [116] showing that the injection of AAV-5 into the cerebellar cortex of mice resulted in extensive dissemination of the virus beyond the injection site. The transduction profile of rAAV1 vectors has also been assessed following direct injection into the CNS of mice [75,117] and *Felis catus* [98], demonstrating higher transduction efficiency with this serotype than with rAAV2, as well as pronounced neurotropism. However, transduction profiles may differ depending on the target brain region [75]. NAbs that reduce AAV2 CNS transduction may be overcome using rAAV vectors based on AAV5, as it is the most non-homologous to AAV2 of all existing serotypes [113].

The evaluation of the in vivo transduction profiles of rAAV7 and rAAV8 vectors revealed higher transduction efficiency compared to rAAV2 vectors in many tissues, including liver and muscle tissues. AAV7 demonstrated a remarkable ability to efficiently transduce skeletal muscle cells, and it also showed levels of transgene expression equivalent to those observed with AAV1, which is known for being the most efficient AAV for transducing skeletal muscle cells. On the other hand, AAV8 showed a strong potential for liver transduction. Research has reported that AAV8 can achieve levels of transgene expression 10- to 100-fold higher than other serotypes (1, 2, 5 and 6). AAV2’s effectiveness has been limited by the low expression level of its transgene in some tissues due to its dependency on certain receptors, which are not ideal for many cell types. In contrast, AAV7 and AAV8 utilize various receptors during the cell entry process, which gives them a great advantage in overcoming AAV2 limitations. Another plus for these two vectors would be their low immunogenicity compared to AAV2 [118,119,120,121]. More recently, Harding et al. examined recombinant AAV vectors pseudotyped with viral capsids derived from AAV serotypes 7 and 8 and assessed their transduction capacity into mouse striatal cells in comparison with vectors pseudotyped with AAV serotypes 2, 5 and 6. The study showed that pseudotyped AAV7 and AAV8 vectors had increased transduction efficiency into the mouse CNS in the rank order rAAV7 > rAAV8 > rAAV5 > rAAV2 = rAAV6, with all vectors demonstrating strong tropism for neuronal transduction. The obtained data demonstrate that rAAV vectors pseudotyped with capsids derived from AAV serotypes 7 and 8 can provide more efficient gene transfer into the CNS [122].

AAV9 is known to transduce approximately twice as many neurons as astrocytes throughout the adult rodent CNS. Co-administration of mannitol had only a moderate effect on CNS transduction, suggesting that AAV9 crosses the BBB via an active transport mechanism. However, when this approach was applied to young primates at the mid-dose tested in mice, it showed reduced transduction of peripheral organs and brain compared to mice, a clear shift towards predominantly glial transduction, and the presence of low levels of pre-existing NAbs largely blocked CNS and peripheral transduction [123].

Studies have shown differences in the transduction profiles of six commonly used AAV serotypes (AAV1, AAV2, AAV5, AAV6, AAV8, AAV9) in different regions of the mouse brain. Despite a common ability to transduce the main brain cell types, expression levels varied for different serotypes and cell types. Notably, rAAV8 was found to be particularly effective in transducing astrocytes, whereas rAAV9 was most suitable for transducing cortical neurons [123,124].

Epilepsy is characterized not only by the aberrant functioning of neurons but also by the extensive involvement of glial cells in the formation and persistence of the pathological process [125]. Therefore, one possible strategy may be to target not only neurons of the brain but also glial tissues [126].

The route and site of vector administration play an important role in determining its transduction and expression capabilities. AAV9-egfp and AAV9-fLuc delivery via intrastriatal (IST), intracisternal (ICM) and lumbar intrathecal (LIT) routes was evaluated in adult rats. The results showed that IST administration provided robust transgene expression in the striatum, thalamus and cortex, with lower transduction in peripheral tissues compared to ICM or LIT administration. ICM administration provided strong expression in more brain regions and similar expression in the spinal cord compared to LIT administration. LIT administration showed lower expression in the brain compared to ICM administration. A similar study was conducted for AAV5. The results showed that IST administration provided centralized localized vector distribution and expression in the frontal part of the brain. Intrathalamic injection demonstrated transduction and gradient expression from the rostral brain to the lumbar spinal cord. Intracerebroventricular administration resulted in more uniform, albeit relatively superficially distributed, transduction and expression throughout the central nervous system. Thus, the choice of route and site of vector administration significantly influences its efficacy and expression distribution, which must be taken into account when developing gene therapies and other biomedical applications [127].

A study by Foust et al. described the intravenous delivery of AAV9 to mice, which effectively affects the brain, dorsal root ganglia, and spinal motor neurons in neonatal animals and astrocytes of the brain and spinal cord of adult mice. This study demonstrated a direct dependence of AAV9 tropism on the age of the animal injected with the vector; there was widespread transduction of neurons, motor neurons in neonatal mice in particular, and extensive transduction of astrocytes in adult mice after intravenous delivery [128]. The ability of AAV9 to penetrate the BBB has also been demonstrated in a number of other studies [123,129]. Directed evolution of this serotype led to the emergence of AAV-PHP.B, which also penetrates the BBB [130]. The high potency of the recombinant serotype was demonstrated in preclinical studies in a synucleinopathy model [131]. A drug was also approved in the European Union and the UK in 2022 for the correction of aromatic L-amino acid decarboxylase (AADC) deficiency using AAV vector therapy [132]. To date, several AAV gene therapies have been approved worldwide. The first AAV2-based drug is Glybera, approved by the European Commission in 2012 for the treatment of lipoprotein lipase deficiency [133]. One of the commercially successful drugs is a drug for the treatment of spinal muscular atrophy (SMA). It is a vector based on the AAV9 virus. Intravenous administration of the drug has been shown to improve motor skills in patients and reduce clinical manifestations of the disease [134]. There is also a gene therapy product Hemgenix, which was approved in 2022 by the Food and Drug Administration (FDA) and is a recombinant AAV5 containing a highly active Paduan variant of the human factor 9 (*F9*) gene with a codon-optimized nucleotide sequence [135].

Thus, the development of AAV vectors for the delivery of genetic material to the epileptic brain must take into account a number of factors, including the type of cells and regions of the brain, the serotype of the virus used, the selection of an appropriate promoter and the route of administration. Although the data on the efficient transduction of brain tissue are encouraging, the tropism and efficiency of the vector may be significantly reduced when used in animals evolutionarily closer to humans. Vector tropism may vary depending on the type and site of administration, as well as the physiological characteristics of the recipient. Although there are no data on the pathogenicity of AAV, its efficiency may be reduced by the presence of antibodies, as well as by improper capsid construction or promoter use.

The ability of AAV to transfer and induce the expression of the *lacZ* marker gene in brain slices from patients undergoing temporal lobectomy was assessed. The AAV-lacZ vector was introduced into brain slices and incubated for 24 h. The expression of the *lacZ* gene was observed after 5 h and was maintained until the end of the experiment, predominantly in neurons, without signs of cytotoxicity. The results confirm the efficiency of AAV in gene transfer in the human CNS. The replacement of the *lacZ* gene with a functional gene may allow localized genetic intervention in focal seizures using stereotactic or endovascular delivery [101].

**Figure 1:**
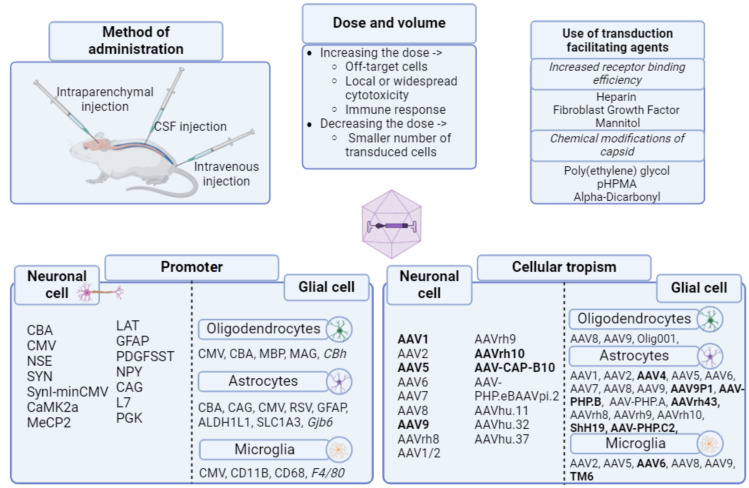
Components of the effective use of AAV vectors in the treatment of CNS disorders.

The development of a suitable AAV vector for the treatment of epilepsy is a complex and multi-step task that requires consideration of all the above factors. These components are essential for the efficient transduction of brain tissue and the successful treatment of epilepsy using AAV gene therapy. The components for the effective use of AAV vectors are summarized in Figure 1.

## 3. Pathogenesis of Epilepsy

The mechanism of epilepsy development consists of the formation of an epileptic focus, the formation of epileptic systems in the brain and brain epileptization. Seizures occur due to under- or over-activation of neurons, and this results in the inability of the brain to coordinate the rest of the human body. Epilepsy can affect one area of the body (focal epilepsy) or the entire body (generalized epilepsy), depending on the location of the affected neurons in the brain [136]. Epilepsy occurs due to genetic susceptibility, development of the nervous system, cerebrovascular factors and other acquired factors that irritate nervous tissue. In addition, it can occur due to neoplasms or metabolic or neurodegenerative disorders, especially among the elderly.

The molecular mechanisms of epileptogenesis are complex and not fully understood but are thought to involve an imbalance between excitatory (glutamate, aspartate) and inhibitory (GABA, taurine, glycine, norepinephrine, dopamine, serotonin) neurotransmitters. A seizure occurs when there is a decrease in inhibitory signaling, such as GABA, or an increase in excitatory signaling, such as glutamate [137,138]. The accumulation of glutamate leads to the degeneration of glutamate receptors, the activation of Na^+^ and Ca^2+^ channels, and the accumulation of Na^+^ and Ca^2+^ ions inside the cell and K^+^ ions in the extracellular fluid. This, in turn, promotes the release of Ca^2+^ from the intracellular depot and the activation of enzymes (phospholipase, protease, etc.), the accumulation of arachidonic acid, increased lipid peroxidation and the destruction of cell membranes [139]. In contrast, GABA-A receptors (ligand-dependent Cl^−^ ion channels) mediate fast inhibitory presynaptic potentials by increasing chloride influx, and GABA-B receptors (G-protein-coupled receptors) mediate slow inhibitory presynaptic potentials by increasing potassium conductivity and decreasing calcium entry [140]. It is assumed that a decrease or loss of GABAergic inhibition may increase the probability of generating excitatory postsynaptic potentials and synchronization volley discharges and, consequently, cause epileptogenesis [141].

Other causes of epileptic seizures are changes in ion concentrations and dysfunction of ion channels—channelopathies. Ion channels are involved in the generation of electrical currents through ion charges. Cation channels mainly generate action potentials and contribute to neuronal excitability; on the other hand, anion channels are involved in the mechanism of inhibition of the neuronal excitatory process, and, thus, ion imbalance can cause epileptogenesis [142]. Mutations in genes expressing potassium, sodium, chloride, calcium channels and acetylcholine and GABA receptors have been reported in epilepsy [143,144]. In addition, channelopathies can also be the pathogenesis of acquired epilepsy due to secondary changes in ion channels through transcriptional and post-translational mechanisms [144].

Inflammation and impaired immune regulation may also play a role in triggering an epileptic seizure. Inflammatory cells release molecules that can alter neuronal signaling, which may lead to seizures. Following seizures, cytokines such as interleukin (IL)-1β, IL-6 and tumor necrosis factor (TNF-α) are released, modulating inflammatory responses in the brain. Studies show that these cytokines influence N-Methyl-D-aspartic acid (NMDA) receptors, synaptic plasticity, GABAergic neurotransmission and neuronal excitability, contributing to seizure development and recurrence [145]. During the epileptic phase, studies of synaptic protein expression, brain inflammation and hippocampal neurogenesis in adult synapsin 2 null mice showed elevated levels of IL-6 and TNF-α [146]. Currently, CNS inflammation caused by BBB permeability is associated with the induction and progression of epilepsy.

## 4. Gain of Function Using an AAV Vector in the Treatment of Epileptic Disorders

One of the key directions in the treatment of epilepsy is gene therapy, which is a promising method for correcting the deficiency or absence of a gene in the body, as well as enhancing its expression to increase the production of the necessary protein. As part of gene therapy, researchers are exploring various strategies for delivering genetic material aimed at restoring normal cell function and reducing the frequency of epileptic seizures. This can be accomplished through the introduction of specific vectors aimed at modulating the operation of ion channels or other molecular components responsible for regulating neuronal excitability. In addition, methods for delivering neuropeptides and neurotrophic factors that can modulate neuronal activity and promote neuronal survival are being actively studied, as well as antisense oligonucleotides, leading to the repression of target genes.

One approach to reducing the frequency and severity of epileptic seizures is to deliver genetic material capable of correcting the lost and/or reduced function of a particular protein product to a cell. The genetic material delivered by the vector construct can encode various functional products, in particular neuropeptides, ion channel subunits, cellular receptors, transcription factors, etc.

### 4.1. Neuropeptides

Most of the studies are aimed at modulating seizure activity by modifying the level of neuropeptides in neuronal tissue. Neuropeptides are messenger molecules that mediate neuronal communication and intercellular signaling in association with neurotransmitters [147]. Indeed, it is known that neuropeptides can regulate inhibition and excitatory processes by repressing the release of glutamate induced by membrane depolarization, which provides an antiepileptic effect [148,149,150].

In existing studies aimed at modulating seizure activity using the AAV vector cassette, neuropeptide Y (NPY) was most often delivered. A study using rAAV2-NPY was conducted on rats exposed to kainic acid. The vector was injected into the rat hippocampus in a volume of 3 μL at a concentration of 1.06 × 10^12^ particles/mL. *NPY* overexpression was restricted to the hippocampus and was observed only in neurons. Peak expression was observed at 2 weeks and persisted for at least 3 months. Immunohistochemical staining revealed *NPY* immunoreactivity in the molecular layer of the hippocampus but not in CA1 and CA3 pyramidal cells or granule neurons. *NPY* mRNA expression was also observed in interneurons and granule cells, as well as in CA1-CA3 pyramidal cells. Applying this viral vector resulted in a reduction in seizure frequency; meanwhile, transduction with the AAV1/2 serotype showed a greater reduction in discrete seizures [151]. In the work of Noe et al., a chimeric rAAV1/2 vector (expressing both serotype 1 and 2 capsid proteins, with an AAV1, AAV2 ratio of 1:1) expressing the *NPY* gene was applied to a model of progressive and spontaneous seizures of temporal lobe epilepsy induced by the electrical stimulation of the temporal pole of the hippocampus. In the experiment, 3 μL of rAAV1/2-NPY with a concentration of 5.2 × 10^12^ particles/mL was injected into four areas of the hippocampus of rats with early epilepsy; additionally, 0.5 μL of heparin was administered. As a result, the introduction of rAAV1/2-NPY into the rat brain led to a significant decrease in seizure progression compared to the control groups. The effect of reducing seizure progression correlated with an increase in *NPY* expression in the hippocampus. The frequency of spontaneous seizures was significantly reduced in 40% of experimental animals compared to the baseline level before injection [152]. Furthermore, with intrahippocampal administration of rAAV1-NPY with the CBA promoter, a significantly better anticonvulsant effect was shown compared to AAV1/2 [153].

Studies have also shown that in addition to NPY, its receptors (Y2) play an important role in the antiepileptic action [154,155,156]. In addition, it was noted that in resected epileptic tissues of patients with epilepsy [157] and rodents [158], a decrease in the expression of Y1 receptors is observed, while the expression of Y2 receptors increases. These data suggest an increase in the effectiveness of the antiepileptic effect of NPY, which is an agonist of the Y2 receptor. In fact, the intracerebral delivery of the agonist NPY13-36 with affinity for the Y2 receptor reduces the predisposition to epileptic seizures after the systemic administration of kainic acid [159]. The truncated neuropeptide fragment NPY13-36 is the C-terminal peptide fragment of NPY, which primarily activates the Y2 receptor, which mediates anticonvulsant activity. When AAV2-NPY or AAV2-NPY13-3 was injected into the piriform cortex of rats, no seizures were observed after kainic acid administration; only control animals developed seizures for 90 min. Additionally, 3/7 rats receiving AAV2-NPY and 1/7 rats receiving AAV2-NPY13-36 developed seizures during the entire observation period [160].

Additionally, studies in rats using rAAV vectors aimed at the overexpression of the Y2 receptor in the hippocampus have demonstrated seizure suppression effects. The combined overexpression of Y2 and NPY showed a more pronounced effect in reducing seizure activity [161].

Gene therapy with AAV1/2-NPY/Y2 in a post-intrahippocampal rat model of chronic epilepsy induced by kainic acid demonstrated not only the prevention of the progressive increase in seizure frequency in treated animals compared to controls but even a 45% reduction in seizure frequency in 80% of epileptic animals [162].

It was also tested as to whether the delivery of the human NPY gene to the thalamus or somatosensory cortex of rats using chimeric AAV1/2 could induce sustained seizure suppression in rats with genetic absence epilepsy. Three cohorts of rats were injected with rAAV-NPY bilaterally into the thalamus or somatosensory cortex. The vector-mediated overexpression of NPY in the thalamus and somatosensory cortex of the genetic absence epilepsy rat from Strasbourg (GAERS) led to a significant reduction in time of convulsive activity and number of seizures; however, a reduction in seizure duration was only seen when the vector was administered into the thalamus. The expression of both human and rat NPY receptors was significantly increased in the somatosensory cortex [163].

More recent studies have also explored the seizure suppression potential of combinatorial gene therapy using an AAV1-based vector carrying the NPY gene and the Y2 receptor in rats. A preliminary study of dose–effect correlation was conducted in a model of systemic acute seizure induced by kainic acid, which revealed the optimal vector titer of 1 μL in three hippocampal sites at a concentration of 10^12^ particles/mL. The vector construct was then administered intrahippocampally in a model of spontaneous recurrent seizures (SRSs). The response rate to therapy was 31% (more than 50% reduction in SRS frequency), and the seizure freedom rate was 13%, while no such effects were observed in control animals. An increase in the intervals between SRSs and a decrease in the duration of SRSs themselves were also observed in the experimental group [164]. Wickham et al. infused the AAV-NPY vector into resected hippocampal tissue slices in vitro for 48 h. Whole-cell patch-clamp analysis of the dentate gyrus (DG) preparation of the hippocampus demonstrated a strong inhibitory effect of NPY on epileptiform activity. In particular, a decrease in the number of paroxysmal depolarizing shifts and action potentials was recorded, which was mediated by Y2 receptors since the use of a selective Y2 antagonist blocked the action of NPY [165].

**Figure 2:**
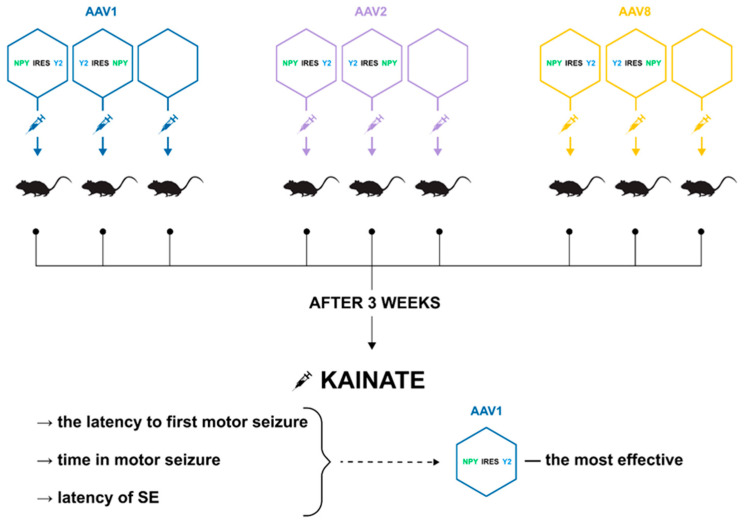
Experimental design for evaluating the efficacy of AAV vectors (serotypes 1, 2 and 8) carrying neuropeptide Y (NPY) and its receptor Y2 in two different transgene orders (NPY/Y2 or Y2/NPY); empty viral cassettes were used as a control. Wistar male rats were injected bilaterally with the viral vectors, followed by subcutaneous kainate injection to induce seizures. Key measurements included latency to the first motor seizure, time spent in motor seizure and latency to status epilepticus. Results from these measurements were used to assess the seizure-suppressant efficacy of the AAV vectors compared to an empty cassette control vector.

The internal ribosome entry site (IRES) sequence separating two gene sequences, such as those for the NPY and Y2 receptor, reduces the translation of the downstream gene, which may also have implications for the potential anticonvulsant effect. Thus, a comparative analysis of three serotypes (AAV1, AAV2 and AAV8) and two transgenes, NPY-IRES-Y2 and Y2-IRES-NPY, in a rat model of acutely induced seizures showed that AAV1-NPY-IRES-Y2 was more effective than other serotypes or transgene sequences, considering both transgene expression and the ability to suppress induced seizures in rats (Figure 2). The vector also demonstrated a transgene-induced decrease in glutamate release from excitatory neuron terminals and significantly increased NPY and Y2 expression in resected human hippocampal tissue from patients with drug-resistant temporal lobe epilepsy [166].

In particular, the combined induction of neuropeptides and their receptors can influence the influx of Ca^2+^ through a decrease in the opening of voltage-dependent calcium channels [167]. Galanin (GAL), for example, is a modulator of neurotransmitter levels in the CNS and peripheral nervous system (PNS) [168] and has been shown to hyperpolarize brain neurons through the opening of K^+^ channels [169,170], as well as through the inhibition of adenylate cyclase activity [171], the inhibition of voltage-dependent Ca^2+^ channels [172] and the regulation of the release of dopamine [173], acetylcholine [174] and glutamate [175].

In the case of NPY, there is evidence that prolonged and persistent epileptic activity can lead to the depletion of neuropeptide-storing vesicles, which contributes to a decrease in the release of this neuropeptide [176]. The seizure threshold is reduced in preprodynorphin (pDyn) knockout mice, leading to an increased susceptibility to developing epilepsy [177]. Similarly, low dynorphin levels in humans are correlated with increased vulnerability to this disease [178,179]. Therefore, the introduction of transgene copies is a relevant therapeutic strategy for reducing seizure activity.

The excitatory inhibitory effect of neuropeptides has been demonstrated in a number of studies; in particular, GAL is able to inhibit the release of acetylcholine in the striatum of the brain and hypothalamus [180], and GAL is also able to repress the release of glutamate and aspartate in the hippocampus [175]. Ultimately, efforts to use neuropeptides in the treatment of epilepsy are aimed at reducing the excitatory activity of brain neurons.

In the work of Haberman et al., cells were transduced in vitro and in vivo with an AAV vector carrying the gene for the galanin neuropeptide *GAL* (AAV-GAL) and also carrying the *GAL* gene and the fibronectin secretory signal sequence *FIB* (AAV-FIB-GAL), which promotes the secretion of galanin from the cells. In vitro, HEK293 cells transfected with AAV-FIB-GAL demonstrated significant galanin content in the medium 24 h after transfection (32 ng/mL), whereas cells transfected with AAV-GAL showed no evidence of secreted galanin [181]. For the in vivo study, a rat model of focal seizures in the lower collicular cortex induced by brief electrical stimulation was used. As a result of the introduction of AAV-FIB-GAL, an increase in the seizure stimulation threshold was observed after 4 weeks. A protective effect was also found after injection of AAV-FIB-GAL into the hilar region of the hippocampus in the kainic model of epilepsy, resulting in the preservation of neurons at a distance of up to 500 μm from the infusion site. Real-time polymerase chain reaction (RT-PCR) demonstrated the stability of galanin expression 4 months after injection of AAV-FIB-GAL into the hippocampus. The work of Lin et al. confirms these results. The administration of an AAV2-GAL-based vector also led to a decrease in the duration of seizure activity in the rats’ injected hippocampus, where galanin was detected in granule cells and DG interneurons of the hippocampus [182]. AAV-FIB-GAL was also infused laterally into the piriform cortex of rats (2 μL, 8 × 10^12^ particles/mL). Following the infusion, experimental rats received a 10 mg/kg dose of kainic acid. Bilateral infusion of AAV-FIB-GAL significantly attenuated kainic acid-induced seizures so that 11 of 12 rats did not exhibit limbic seizures, and one rat exhibited only a brief, single class III seizure. AAV-FIB-GAL infusion prevented electrographic seizure activity. In contrast, bilateral infusion of AAV-FIB-GFP did not alter either behavioral or electrographic seizure activity. Since prior seizure exposure could affect the efficiency of the transporter, another group of rats received daily electrical stimulation of the piriform cortex until three consecutive class V seizures occurred. AAV-FIB-GAL or AAV-FIB-GFP (3 μL/30 min) were then injected into the electrode site. After one week, AAV-FIB-GAL rats showed a significant increase in the stimulation current required to elicit limbic seizure activity, while AAV-FIB-GFP did not alter the seizure threshold. Thus, AAV-mediated galanin expression and secretion significantly suppresses limbic seizure activity in vivo [183].

Gene therapy with AAV encoding preprodynorphin (pDyn) has been shown to suppress seizures for several months in mice and rats with an epileptogenic model of temporal lobe epilepsy. Preprodynorphin is a precursor of dynorphins. Dynorphins are a family of endogenous opioids stored in vesicles that are recognized as natural anticonvulsants [184]. AAV1-pDyn and AAV1-GFP at a dose of 2 × 10^9^ genomic particles (gp) were administered into the epileptogenic lesion approximately 1 month after KA injection when focal epilepsy developed. Delivery of AAV1-pDyn caused a gradual decrease in the frequency of generalized seizures. Generalized seizures completely disappeared within 1 week, and no further seizures were observed during the entire observation period (3 months). In contrast, animals treated with AAV1-GFP continued to have seizures throughout the observation period. To demonstrate that Dyn’s action is mediated by kappa opioid receptors, mice were injected with the KOR antagonist norBNI (20 mg/kg) 30 days after AAV1-pDyn delivery, at which time seizures had disappeared. Drug treatment resulted in a transient resumption of seizures, which disappeared when the antagonist was washed out.

### 4.2. Ion Channels, Receptors and Membrane Proteins

Another direction is the delivery of ion channel subunits or membrane receptors designed to compensate for insufficient function or reduce excessive function. The dysfunction of ion channels can promote the propagation of excitation and/or the disruption of inhibitory processes, which ultimately lead to the occurrence of seizure activity [185]. Work in this orientation can go both in the direction of enhancing the function and in the direction of decreasing it.

Ion channels are transmembrane proteins that are selective pores for ions that regulate neuronal excitability [185]. The induction of ion channel function, as well as specific cellular receptors, has also been one of the goals in a number of studies. In particular, a decrease in the function of the local NMDA receptor can reduce sensitivity to epileptic seizures [186]. Oral vaccines are taken up by intestinal M cells, are rapidly taken up by antigen-presenting cells (APCs) in Peyer’s patches and lamina propria, and can induce potent humoral immune responses. The use of an oral AAV vaccine targeting the NR1 subunit of the NMDA receptor resulted in the production of an autoantibody and a potent humoral response to NR1, which contributed to potent antiepileptic and neuroprotective activity in both the KA model of epilepsy and the stroke model of rats [187].

The *SCN1A* gene encodes the alpha subunit of the voltage-gated sodium channel type I (NaV1.1) [188]. NaV1.1 is predominantly expressed in the axon initial segment of GABAergic inhibitory interneurons, where it generates and propagates action potentials [189]. The genetic reduction of NaV1.1 significantly reduces the frequency and amplitude of action potentials generated by GABAergic inhibitory interneurons, thereby impairing their inhibitory function [190,191,192].

In an analysis of the therapeutic efficacy of *SCN1A* gene regulation using AAV9, AAV9-RE^GABA^-eTF*^SCN1A^* was designed to activate the *SCN1A* gene from the endogenous genome since the *SCN1A* gene exceeds the packaging capacity of AAV, which expresses an engineered transcription factor (eTF*^SCN1A^*) designed to upregulate the *SCN1A* gene from the endogenous genome. A cell-selective regulatory element (RE^GABA^) was included to target transgene expression specifically to GABAergic inhibitory interneurons. A single bilateral intracerebroventricular administration of AAV9-RE^GABA^-eTF*^SCN1A^* to experimental mice at postnatal day 1 resulted in an increase in *SCN1A* mRNA transcripts, especially in GABAergic inhibitory interneurons, and NaV1.1 protein levels in the brain. A significant reduction in spontaneous and hyperthermic seizure frequency, as well as an increase in survival, was demonstrated. In non-human primates (NHPs), the delivery of AAV9-RE^GABA^-eTF*^SCN1A^* via unilateral intracerebral injection resulted in widespread biodistribution of the vector and transgene expression throughout the brain, including key structures involved in epilepsy, such as the hippocampus and cerebral cortex, with no adverse effects, no noticeable changes in clinical observations, no adverse histopathological findings and no dorsal root ganglia-related toxicity [193].

At the same time, enhancing the function of certain types of receptors and ion channels can also lead to a reduction in seizure activity. For example, the endocannabinoid system is considered a therapeutic target in epilepsy [194]; therefore, effective treatment strategies that exploit CB1 receptor regulation require a detailed understanding of the effects of the CB1 receptor in neuronal subtypes. To this end, the analysis of conditional mutant mice lacking CB1 receptors on different neuronal subtypes subjected to kainic acid (KA)-induced seizures showed that CB1 receptors on glutamatergic but not GABAergic hippocampal neurons are required for protection against excitotoxic seizures [195]. In line with these preclinical data, specific reductions in CB1 receptor protein and mRNA levels at glutamatergic but not GABAergic axon terminals have been reported in human epileptic hippocampal tissue [196]. However, these conditional loss-of-function studies have not yet been complemented by a corresponding gain-of-function approach involving CB1 overexpression, thereby precluding a full picture of CB1-mediated control of hyperexcitability. Also, potassium channels are considered promising tools for gene therapy due to their remarkable ability to hyperpolarize neurons and thus suppress their activity [197]. Most genetic approaches to suppress seizures utilize the expression of potassium channels or other potassium conductance-related proteins [198,199,200,201]. Moreover, many forms of epilepsy are known to be correlated with loss of potassium channel function [202,203,204,205,206]. Voltage-gated Kv1 channels are involved in the generation of action potentials (APs) [207,208]. The duration of APs controls the amplitude of evoked postsynaptic potentials [209,210], which affects downstream signal transmission.

Studies in humans with temporal lobe epilepsy (TLE) and in rodent models of TLE have found decreased expression of the GABA receptor GABR1 subunit and increased expression of GABR4 subunits in the DG of humans with epilepsy [211]. These changes begin within 24 h of pilocarpine-induced status epilepticus (SE) in adult rats and persist for several months as these animals gradually develop epilepsy [212]. Rats that experienced pilocarpine-induced SE on postnatal day 10 had increased GABR1 expression in the DG and enhanced benzodiazepine-induced increases in GABA type I currents, and none developed epilepsy later in life [213].

Raol et al. used AAV5 containing GABRA4 promoter to transduce the DG after induced status epilepticus (SE). They detected an increase in GABRA4 subunit mRNA and protein expression in the DG 1–2 weeks after SE. Increased GABRA4 expression in the DG resulted in a three-fold increase in the mean seizure-free time after induced SE and a 60% decrease in the number of rats that developed epilepsy (recurrent spontaneous seizures) in the first 4 weeks after SE [214].

Nikitin et al. [215] reported an AAV vector designed to reduce epileptiform activity by expressing the human Ca^2+^-gated K^+^ channel *KCNN4* gene for a calcium-activated potassium channel, KCa3.1, to target virus expression to glutamatergic excitatory pyramidal neurons, which make up the majority of the cerebral cortex. AAV2-KCNN4-Venus, driven by the CaMKII promoter, was injected into adult mouse brains (2 × 10^10^ vg/μL). The results showed that KCNN4-transduced cells exhibited Ca^2+^-dependent slow afterhyperpolarization, which significantly reduced the ability of KCNN4-positive neurons to generate high-frequency spike trains without affecting their low-frequency coding capacity and action potential shape. Antiepileptic activity tests showed potent suppression of pharmacologically induced seizures in vitro at both single-cell and local field levels, with a reduction in peaks during ictal discharges.

AAV1/2-CB1 was injected into the hippocampus of adult mice to evaluate the cannabinoid receptor CB1—the most abundant G protein-coupled receptor in the brain and a key regulator of neuronal excitability—and to investigate the effects of increasing the *CB1* gene dosage in the hippocampus on the development of epileptiform seizures and neuronal injury in the kaine epilepsy model. Transgene expression was restricted exclusively to Cre-recombinase-expressing principal neurons using an AAV expression cassette with a transcription stop cassette flanked by loxP sites preceding the transgene. Collectively, it has been demonstrated that the ectopic CB1 receptor is highly expressed by cell type, particularly in hippocampal pyramidal neurons, localized to presynaptic sites and coupled to G proteins. The severity and mortality of KA-induced seizures were reduced in CB1 receptor overexpressors compared to AAV-treated controls. Neuronal damage in the CA3 region of the hippocampus is notably absent in AAV-treated Cre transgenic animals but is evident in all cortical regions of both treatment groups [216].

Neuroligins 2 (NL2) is selectively found at inhibitory synapses. The interaction between presynaptic neurexin and postsynaptic NL2 enhances inhibitory synaptic transmission. NL2 binds GABA receptors in the scaffold in the postsynaptic neuron through molecular interactions with gephyrin and collybistin, and the global induction of NL2 may alleviate generalized seizures. The application of AAV9 to express *NL2* resulted in a significant reduction in seizure duration, severity and frequency in an epilepsy mouse model. Exogenous *NL2* was expressed widely in the brain and co-localized with the postsynaptic inhibitory molecule gephyrin [217].

Collectively, these studies demonstrate the high potential of gene therapy using different vectors and genes to effectively control epileptic seizures. These approaches open new horizons in the development of personalized and targeted therapies for epilepsy, providing significant improvement in the quality of life of patients.

### 4.3. Neurotrophic and Transcription Factors

Neurotrophic factors are peptides or small proteins involved in the growth, differentiation, development and survival of neurons. Brain-derived neurotrophic factors are involved in the pathogenesis of epilepsy and may have a neuroprotective effect [218]. The overexpression of rAAV2-based glial cell-derived neurotrophic factor (GDNF) in the rat hippocampus has been shown to suppress seizures in two models of temporal lobe epilepsy [219]. In the case of GDNF, upregulation of GDNF messenger RNA and protein has been demonstrated in the granule cell layer (GC) of the DG and hilus, as well as in the pyramidal layer of the hippocampal cornu ammonis (CA1–CA3) regions, after induction of status epilepticus with kainic acid [220,221] and lithium-pilocarpine [222], suggesting the involvement of GDNF in the epileptogenic process. Most importantly, KA-induced generalized tonic–clonic seizures [223] and kindling-induced epileptogenesis are suppressed by intraventricular infusion of GDNF [224]. Based on these data, it could be hypothesized that *GDNF* gene transfer to the epileptogenic focus would have an inhibitory effect on both epileptogenesis and the occurrence of generalized seizures. In line with the latter concept, adenoviral vector-mediated overexpression of GDNF in the hippocampus has been shown to suppress KA-induced generalized tonic–clonic seizures [225].

Also, the delivery of transcription factors seems promising. For example, Nrf2 regulates the expression of a number of genes encoding detoxifying, antioxidant and anti-inflammatory mediators; calcium homeostasis regulators; and signaling molecules, which leads to an organized protective response. Genes predominantly regulated by Nrf2 include those encoding heme oxygenase-1 (HO-1), NAD(P)H quinone oxidoreductase 1 (NQO1), microsomal glutathione S-transferase (MGST), glutathione peroxidase and superoxide dismutase [226,227,228], which may provide a cascade of protective events.

A study by Mazzuferi demonstrated that the overexpression of the transcription factor Nrf2, which promotes the expression of numerous antioxidant, anti-inflammatory and neuroprotective proteins, using AAV can reduce the incidence of spontaneous recurrent seizures (SRSs) and preserve neurons in the hippocampus. Following the administration of AAV-Nrf2 into the hippocampus of mice, mRNA levels of *Nrf2* and related genes (*HO-1*, *NQO1*, and *MGST*) increased, peaked 72 h after the seizure episode and then decreased. Mice receiving AAV-Nrf2 showed a significant reduction in the number of generalized seizures and decreased microglial activation, while the number of neurons was preserved and the number of astrocytes was not affected [229].

### 4.4. Other Transgenes for Delivery

In addition to neuropeptides and channels, other genes have been investigated for targeting delivery to the CNS. A study by Klugmann et al. examined the role of overexpressing different domains of the Homer 1 protein in the hippocampus of adult rats using rAAV. Homer 1c has both a ligand-binding domain and a coiled-coil domain for self-multimerization, but Homer 1a lacks the coiled-coil domain. In this study, a new isoform, 1g, which lacks the ligand-binding domain of Homer, was analyzed. AAV vectors were generated expressing Homer 1a, 1c and 1g under the control of the CBA promoter. The viral vectors were injected into the rat hippocampus. Histological analysis revealed no gross brain abnormalities 4 weeks after vector injection. To evaluate the effect of AAV-Homer 1a overexpression, a model of self-sustaining limbic status epilepticus (SSLSE) induced by focal hippocampal stimulation was used. In rats treated with AAV-Homer 1a, SSLSE was strongly suppressed, in contrast to Homer 1c, Homer 1g and control groups, where SSLSE was successfully induced. During SSLSE, control and Homer 1c and 1g rats exhibited intense motor seizure activity, including clonic seizures and forelimb raising [230].

Another way to reduce excitatory activity and excitotoxicity is to deliver genes responsible for glutamate metabolism. Glutamate transporters are a family of neurotransmitter carrier proteins that transfer glutamate, the major excitatory neurotransmitter, across the membrane. Transporters play an important role in regulating the concentration of glutamate in the extracellular space by transporting it, along with other ions, across cell membranes [231]. Changes in the expression of excitatory amino acid transporter 2 (EAAT2) and glutamine synthetase (GS) have been detected in sclerotic hippocampal tissue removed during epilepsy surgery [232,233,234,235], and the finding that elevated extracellular glutamate levels appear to trigger spontaneous seizures in humans with TLE [236] has provided some impetus to determine whether these proteins would be suitable targets for gene therapy. However, whether glutamate transporters are altered in human epilepsy remains controversial; two studies found no changes in transporter expression in resected tissue from TLE patients [237,238], whereas region-specific changes in EAAT1-3 expression have also been reported [233,235,237].

GS in the brain is involved in the metabolic regulation of glutamate, brain ammonia detoxification, ammonia assimilation, neurotransmitter recycling and neurotransmitter signaling termination [239,240]. GS in the brain is found predominantly in astrocytes [241]. Astrocytes protect neurons from excitotoxicity by scavenging excess ammonia and glutamate [240]. GS expression in astrocytes is also reduced in resected epileptic tissue [235,237].

In another study, the AAV-mediated overexpression of GS and EAAT2 or the expression of adenosine kinase-targeting microRNA (miR-ADK) under the control of the glial GFAP promoter modulated susceptibility to kainate-induced seizures and neuronal cell loss in rat hippocampal astrocytes. After the injection of AAV9 vectors into the rat hippocampus, transgene expression was detected after 3 weeks, predominantly in astrocytes. ADK expression in miR-ADK vector-loaded rats was reduced by 94–96%, which was accompanied by a ~50% reduction in kainate-induced seizure duration and better protection of dentate neurons but not CA3 neurons, compared with controls. In contrast, the infusion of AAV9-GS and AAV9-EAAT2 vectors did not provide protection against seizures or neuronal injury due to the low transcriptional activity of the GFAP promoter, which did not result in a significant increase in transgenic GS or EAAT2 levels [242].

Data on AVV-mediated delivery of therapeutic genes are compiled in Table 1.

## 5. Loss of Function Using AAV Vectors

A less used but nonetheless effective approach in gene therapy is the delivery or expression of antisense sequences. Traditionally, antisense oligonucleotides have been employed, but specific genes can also be targeted using antisense RNAs inserted into an AAV vector cassette [243]. Antisense oligonucleotides are synthetic single-stranded nucleic acid sequences that bind to RNA and thereby alter or reduce the expression of the target RNA. They can not only reduce the expression of mutant proteins by cleaving the target transcript but also restore protein expression or modify proteins by interfering with pre-mRNA splicing [244].

GABA is the major inhibitory neurotransmitter in the brain. In the inferior colliculus and other brain regions, GABA receptors directly modulate seizure susceptibility. Increased GABA-A receptor activity attenuates seizure genesis, while the blockade of GABA-A receptor function increases seizure susceptibility. Thus, an AAV vector with a CMV promoter and truncated human GABA-A-α1 cDNA in both sense and antisense orientations was constructed. Following microinjection of AAV-GABA-A-α1 sense vectors at a dose of 3 × 10^9^ particles/μL, neurons exhibited GABA-A-α-like immunoreactivity significantly exceeding endogenous concentrations after 7 days. The infusion of antisense vector (3 × 10^8^ particles/μL) resulted in increased seizure duration and decreased [3H]zolpidem binding, which may affect seizure susceptibility in vivo [245].

In contrast, the reduction of local N-methyl-d-aspartic acid (NMDA) receptor function may attenuate seizure susceptibility. The work of Haberman et al. was one of the first to explore the approach of blocking specific molecules by delivering antisense sequences using AAV vectors. An NMDA receptor cDNA fragment in the antisense orientation was cloned into AAV vectors (AAV-NR1A) where expression was driven by either tetracycline-off regulatable promoter (AAV-tTAK-NR1A) or a cytomegalovirus promoter (AAV-CMV-NR1A). The in vitro infection of neurons with AAV-tTAK-NR1A resulted in decreased NMDA-evoked currents and a decrease in the number of NMDA receptors, and the intracortical administration of AAV-tTAK-NR1A to rats reduced NMDAR1 subunit protein levels in vivo. However, the administration of AAV-CMV-NR1A caused a significant reduction in seizure sensitivity. The additional administration of AAV-tTAK-GFP and AAV-CMV-LacZ transduced different neuronal populations. The results indicate that promoters can significantly influence the physiological outcome of NMDA receptor-based gene therapy [246].

Adenosine kinase (ADK) is a negative regulator of the endogenous brain anticonvulsant adenosine in astrocytes. ADK-transgenic mice and wild-type mice were injected with the AAV8-Adk vector in sense or antisense orientation under the control of the gfaABC1D promoter to overexpress or downregulate ADK in astrocytes. It was shown that in wild-type mice, overexpression of ADK in astrocytes was sufficient to trigger spontaneous recurrent seizures in the absence of any other epileptogenic events, whereas the downregulation of ADK with antisense AAV8-Adk almost completely abolished spontaneous recurrent seizures in ADK-transgenic mice [247].

Tubulin β-III (TUBB3) is the most dynamic β-tubulin isoform expressed in neurons and is highly expressed in the CNS. *TUBB3* expression has been found to be increased in human and rat epileptic tissues. Moreover, *TUBB3* expression is associated with inhibitory GABAergic neurons and the inhibitory postsynaptic scaffold protein gephyrin. TUBB3 downregulation attenuated seizure behavioral phenotypes during the pilocarpine-induced chronic seizure phase and pentylenetetrazole kindling, whereas TUBB3 overexpression had the opposite effect. Importantly, TUBB3 interacted with GABA-A receptor-associated protein, which is known to be involved in GABA-A receptor trafficking. These results indicate that TUBB3 plays a critical role in the regulation of epileptic seizures through the trafficking of GABA-A receptors, suggesting a molecular mechanism for new therapeutic strategies by AAV delivery of these ion channel subunits [248].

A promising approach is the delivery of antisense nucleotides to block the expression of potentially epileptogenic proteins. In particular, blocking the expression of long non-coding RNA (lncRNA) H19 can prevent its induced apoptosis [249]. LncRNAs are RNA molecules longer than 200 nucleotides that do not have the ability to encode a protein. Individual lncRNAs demonstrate cell-, tissue- and line-specific expression patterns, also indicating the involvement of lncRNAs in the regulation of gene expression at both the transcriptional and translational levels. In addition, lncRNAs are likely to contribute to the development of various diseases, including nervous system disorders. In the study of Han et al., lncRNA H19 was analyzed, which is reactivated in the latent period of epilepsy. Vectors carrying H19 (AAV9-H19) or short hairpin RNA targeting H19 (AAV9-shRNA) were delivered in a KA rat model of epilepsy. According to the functional annotation of genes, many genes inhibited or stimulated by H19 are involved in myelin assembly, immune responses, apoptosis and MAPK activation. The demyelination of nerve fibers, hyperactivation of MAPK and apoptosis of hippocampal neurons are key processes in epileptogenesis. It is indicated that H19 can induce epileptogenesis by inhibiting myelin assembly and inducing the demyelination of nerve fibers. In addition, H19 can both enhance and suppress MAPK activity, which is also associated with epileptogenesis. Functional and pathway analysis reveals that H19 has diverse functions in epileptogenesis, representing potential targets for future studies of H19-modulated mechanisms [250].

Wong et al. used small hairpin RNA (shRNA) directed against the *Scn8a* gene, which resulted in the selective downregulation of *Scn8a* expression in the hippocampus. *SCN8A* encodes the voltage-gated sodium channel (VGSC) Nav1.6, which is widely expressed in the CNS and PNS, where it strongly modulates neuronal excitability by setting the action potential initiation threshold and generating subthreshold depolarizing currents in the soma and dendrites. AAV expressing shRNA against *Scn8a* (shAAV-Scn8a) was injected acutely into the hippocampus of mesial temporal lobe epilepsy (MTLE) mice 24 h after KA administration and was found to arrest the development of spontaneous seizures, attenuate KA-induced hyperactivity and reduce reactive gliosis. Thus, the downregulation of *Scn8a* expression can prevent the occurrence of spontaneous seizures in a mouse model of MTLE [251].

In temporal lobe epilepsy, recurrent mossy fibers sprouting from Dentate Gyrus Granule Cells (DGGCs) create an aberrant epileptogenic network between DGGCs that acts through ectopically expressed GluK2/GluK5 containing kainite acid receptors (KARs). The pharmacological inhibition of KARs or the genetic deletion of GluK2-containing KARs is known to reduce interictal epileptiform discharges [252]. Therefore, an AAV9 vector expressing anti-*grik2* microRNA was designed to specifically downregulate GluK2 expression. The hippocampal delivery of AAV9-anti-*grik2* miRNA was shown to markedly reduce chronic seizure activity in mice with temporal lobe epilepsy [253].

The data on the repression of some genes using AAV vectors are compiled in Table 2.

Thus, there are different approaches to using AAV vectors for the treatment of epileptic disorders. The enhancement of the function of a particular target protein is the most commonly used; however, data suggest that AAV-induced gene knockout may also be quite effective. Unfortunately, most of the data were obtained in animals that are quite evolutionarily distant from humans, so the question of the prospects for clinical trials remains open. Further research in this area would help to select the most effective serotype and promoter for the sufficient transduction of human cells without the occurrence of undesirable effects.

## 6. Conclusions

In this article, we reviewed the use of AAVs in gene therapy for epilepsy, examining their mechanisms of action, advantages and disadvantages. Different AAV serotypes differ in their surface properties, which affects their tropism, ability to penetrate the BBB and the efficiency of cell transduction. This diversity allows for the selection of the optimal serotype for a specific therapy. Combining AAV genomic constructs with capsid proteins of different serotypes creates pseudotyped recombinant AAVs and expands their application to various cell types and tissues. The use of different promoters in vector constructs allows for tissue specificity and maximum efficiency of gene therapy [73].

Studies using AAV vectors in gene therapy for epilepsy demonstrate significant potential in modulating seizure activity by delivering various genes and neuropeptides that can regulate inhibition and excitation processes by repressing the release of glutamate induced by membrane depolarization, which provides an antiepileptic effect. The most successful experiments are associated with the delivery of NPY and its receptor Y2. The overexpression of NPY in the rat hippocampus resulted in a decrease in seizure frequency, and the use of the rAAV1/2-NPY vector successfully suppressed seizures in models of temporal lobe epilepsy. Therapy using AAV1-NPY-IRES-Y2 also showed efficacy in human hippocampal tissue, especially in patients with drug-resistant epilepsy. In addition to NPY, the therapeutic potential of other neuropeptides is being explored, such as galanin, which also increased the seizure threshold and exhibited a protective effect. These results confirm the potential of AAV vectors to modulate neuropeptide systems and develop new treatments for epilepsy [166,181].

Studies have shown that ion channels are promising targets in gene therapy for epilepsy. The introduction of genes encoding various ion channels and receptors, such as GABRA4, SCN1A and KCNN4, using AAV vectors resulted in significant reductions in seizure frequency and severity in preclinical models. Gene therapy targeting the expression of neurotrophic factors and other genes in the central nervous system also represents a promising approach for the treatment of epilepsy. Factors such as GDNF and transcription factors such as Nrf2 have been shown to reduce seizure frequency and protect neurons [214,215,217]. The incorporation of antisense RNAs into vector cassettes allows for the targeted manipulation of the expression of specific genes, such as GABA and NMDA receptors, as well as ADK, which significantly impacts seizure activity [246,247].

Thus, modulating the expression of various neuropeptides, ion channels, transcription factors, etc., can regulate seizure activity and, consequently, reduce the severity of epileptic disorders. Targeting both neuronal and glial proteins may be a promising strategy in treatment. Another important strategy is gene knockout since the cDNA transcript is shorter than the functional gene sequence, which simplifies the use of AAVs since their packaging capacity is small. Additionally, delivering the transcription factors required for inducing target gene transcription appears to be a promising strategy since a number of functional proteins can be under the control of the same transcription factor, which would provide the possibility of multiplex targeting of a number of genes. The study of the proteomic and transcriptomic expression profiles of epileptic tissues and animal models can help in finding potential targets for knockout or enhancement of expression by transgene delivery.

## Figures and Tables

**Table 1:** Data on AVV-mediated delivery of therapeutic genes.

Serotype	Delivery Gene	Model	Antiepileptic Effect	Reference
AAV2; AAV1/2	*NPY*	KA, rats	Reducing the number of seizures; reducing the duration of seizure activity	[151]
AAV2		KA, rats	Increase in the latent period of limbic convulsive activity	[160]
AVV1/2		Electrical stimulation, rats	Reducing the frequency of seizures; decrease in the progression of seizures by 80%	[152]
AAV1		KA, rats	Reducing the number of seizures by 55%; reducing the duration of ictal activity	[153]
AAV1/2		KA, rats	Preventing the progression of the frequency of seizures by 57.5 ± 19.3%; reducing the duration of seizure activity	[162]
AAV1/2		GAERS GGE, rats	Reducing the duration of seizure activity; reducing the number of seizures	[163]
AAV1		KA, rats	Reducing the severity of seizures; increase in the latent time; decrease in the frequency and total time of seizure activity; SRS suppression	[164]
AAV1, AAV2, AAV8		KA, rats + resected human hippocampus	AAV1 reduces the time spent on motor seizures and increases the latency period to SE	[166]
AAV2	*Y2*	Kindling, KA, rats	Reduction of the cumulative degree of seizures; reducing the number of severe grade 4–5 seizures and increasing the amount of stimulation needed to achieve grade 3 or 4–5 seizures	[161]
AAV2	*GAL*	KA, rats, HEK 293 cells	Decrease in in vivo sensitivity to focal seizures and prevention of hilar cell death caused by kainic acid	[181]
AAV2		KA, rats, HEK 293 cells	Increase in the threshold of excitability; reduction of neuronal death	[182]
AAV2		KA, Kindling, rats	Suppression of limbic seizures; increase in the stimulation current required to induce limbic convulsive activity	[183]
AAV1/2	HOMER 1	Electrical stimulation, rats	Suppression of SSLSE	[230]
AAV2	GABRA4	Electrical stimulation, rats	Suppression of SSLSE	[214]
AAV2	*GDNF*	Kindling, rats	Suppression of SSLSE; increased survival after SE	[219]
AAV1/2	CB1	KA, mice	Reducing the severity of seizures; protection from excitotoxicity and neuronal death	[216]
AAV2	*Nrf2*	Lithium-pilocarpine model, mice	Decrease in microglia activation; the ratio between astrocytes/neurons and activated microglia/neurons was significantly lower	[229]
AAV9	*EAAT2*	KA, rats	There were no differences in the total duration of seizures between the animals that were injected with the EAAT2, GS vector, and the control vector	[242]
	*GS*			
AAV1	*Dyn*	KA, electrical stimulation, rats, mice	Suppression of seizures; the disappearance of generalized seizures	[184]
AAV2	*NL2*	Models of polygenic epilepsy, mice	Significant decrease in the duration, strength and frequency of seizures was observed over a 14-week period	[217]
AAV9	*SCN1A*	Scn1a^+/−^, mice, primates	Reducing the frequency, duration and severity of spontaneous seizures; reduces the sensitivity of HTS in Dravet mice	[193]
AAV2	*KCNN4*	4-aminopyridine model, mice	Powerful suppression of pharmacologically induced seizures in vitro both at the level of single cells and at the level of the local field, with a decrease in peaks during ictal discharges	[215]

**Table 2:** The data on the repression of some genes using AAV vectors.

Serotype	Gene for Blocking	Model	Effect	Reference
AAV2	GABA(A)	KA, Kindling, rats	Modulation of colliculi inferiores convulsions	[245]
AAV2	NMDAR1	KA, rats	Significant decrease in sensitivity to focal seizures within 4 weeks. The design of the pTet promoter caused the opposite effect—a significant increase in sensitivity to seizures. It has been shown that in the brain, NMDA receptor excitation can cause GABA inhibition [21]; therefore, removing NMDA receptor excitation from these inhibitory interneurons can create a state of hyperexcitability.	[246]
AAV8	ADK	Transgenic mice with spontaneous epilepsy	Preventing convulsive activity	[247]
AAV9	lncRNA H19	KA, rats	The CA3 neurons were preserved	[250]
AAV10	*Scn8a*	KA, mice	Protection from spontaneous seizures; reduction of gliosis	[250]
AAV9	GluK2	Pilocarpine, mice	Reduction of chronic convulsive activity	[253]

## Data Availability

Not applicable.
